# Correlation and Trends in Primary Health Care and Family Health Strategy Coverage of Leprosy Detection in Minas Gerais

**DOI:** 10.3390/ijerph22040490

**Published:** 2025-03-25

**Authors:** Daniele dos Santos Lages, Isabela Cristina Lana Maciel, Sarah Lamas Vidal, Francisco Carlos Félix Lana

**Affiliations:** 1 Postgraduate Program in Nursing, School of Nursing, Federal University of Minas Gerais, Belo Horizonte 30130-100, Minas Gerais, Brazil; daniele-lages@ufmg.br (D.d.S.L.); isabelaclm@ufmg.br (I.C.L.M.); sarahlamasvidal@ufmg.br (S.L.V.); 2 Department of Maternal and Child Nursing and Public Health, School of Nursing, Federal University of Minas Gerais, Belo Horizonte 30130-100, Minas Gerais, Brazil

**Keywords:** leprosy, epidemiology, public health surveillance

## Abstract

Leprosy, a chronic disease caused by Mycobacterium leprae, continues to be a significant public health challenge in Brazil, which has a high rate of new cases and late diagnoses. This study investigates the relationship between Primary Health Care (PHC) and Family Health Strategy (FHS) coverage and leprosy detection in Minas Gerais, a state marked by heterogeneity in the distribution of the disease. This observational and ecological study analyzed data from 2010 to 2020, which were obtained from the Notifiable Diseases Information System (SINAN) and the EGESTOR-AB portal. Using statistical methods, such as Pearson’s correlation and Prais–Winsten Linear Regression, trends and associations between coverage variables and leprosy indicators were assessed. This study found that PHC and FHS coverage expansion in Minas Gerais was not directly associated with a uniform reduction in late leprosy diagnosis. The findings indicate that the expansion of PHC and the FHS has not been accompanied by a homogeneous reduction in late diagnoses. It is therefore recommended that active surveillance actions be strengthened, that teams be continuously trained, and that strategies recommended by the WHO be integrated.

## 1. Introduction

Leprosy, a chronic disease with insidious progression caused by Mycobacterium leprae, remains a public health challenge in Brazil, a country that ranks among the top countries in terms of the number of annual new cases, and is also notable for its high burden of late diagnosis [1]. Despite advances in leprosy control, complex issues, such as late diagnosis and the persistence of transmission in more vulnerable regions, expose structural and operational limitations in the health network, indicating that health services may fall short of local demands [2].

In the state of Minas Gerais, where there is a heterogeneous distribution of new cases and an endemic pattern in various regions, Primary Health Care (PHC) and the Family Health Strategy (FHS) are fundamental pillars for promoting early diagnosis and continuity of treatment. However, the specific relationship between the coverage of these structures and the capacity to detect leprosy remains little explored, which represents an obstacle to strengthening more effective and equitable health policies throughout the state [3,4].

The Family Health Strategy (FHS) was implemented in Brazil as a health care model that aims to promote health and prevent diseases through integrality and promoting access to health services, especially for the most vulnerable populations [4]. The expansion of the FHS, in particular, has been pointed out in studies as a factor associated with increased detection of infectious diseases, including leprosy, due to the possibility of contact tracing, continuous surveillance, and local action in communities.

Previous studies indicate that Primary Health Care and FHS coverage are associated with improvements in health indicators, but there is still a gap in knowledge about how these variables interact specifically in the context of leprosy [5]. Such disparities between areas of high and low coverage indicate the need to understand whether and how the FHS, in synergy with other components of PHC, responds to the specificities of highly endemic regions.

This research is justified by the need to establish a more detailed understanding of the relationship between PHC and FHS coverage and leprosy detection effectiveness in Minas Gerais, contributing to the formulation of policies that are more focused on regional disparities. Understanding this relationship also allows us to envision strategies to optimize the existing resources and reach communities in highly vulnerable contexts, promoting more appropriate and efficient interventions in leprosy control [6]. In this sense, it is essential to understand whether the expansion of FHS coverage really contributes to improving leprosy detection rates and how this relationship manifests itself in different regions of Minas Gerais.

Thus, the central question of this study is as follows: Does Primary Health Care and Family Health Strategy coverage influence the detection of new leprosy cases in different regions of Minas Gerais? The hypothesis of this study is that the expansion and consolidation of PHC and FHS coverage in Minas Gerais is positively associated with the early detection of leprosy, especially in areas where the disease burden is higher. Therefore, it is expected that regions with greater FHS and PHC coverage will have higher detection rates and a reduced proportion of cases with a degree of disability since easier access and continuous monitoring of vulnerable populations could increase the early identification of cases.

Thus, the aim of this study is to investigate the relationship between PHC and FHS coverage and leprosy detection in the state of Minas Gerais, analyzing temporally the impact of these structures on early detection and leprosy monitoring indicators in endemic areas.

## 2. Materials and Methods

This is an ecological observational study aimed at investigating the relationship between Family Health Strategy (FHS) and Primary Health Care (PHC) coverage and leprosy detection rates in the health micro-regions of the state of Minas Gerais, Brazil, between 2010 and 2020. This study was conducted at the state level and in the health micro-regions of Minas Gerais, a territory with high endemicity for leprosy.

Leprosy data were obtained from the Notifiable Diseases Information System (SINAN), and new cases diagnosed and notified during the study period were analyzed. Only new cases notified from 2010 to 2020 were included, excluding records identified as diagnostic errors in the type of output. Coverage data for Primary Health Care and the Family Health Strategy were obtained from the EGESTOR-AB portal of the Department of Informatics of the Unified Health System (DATASUS). These coverage data were available until 2020, which defined this study’s period of analysis.

In order to assess the relationship between PHC and FHS coverage and leprosy indicators, the following indicators were calculated: the overall detection rate of new leprosy cases (refers to the total number of new cases detected per year) and the detection rate of new leprosy cases with grade 2 disability at the time of diagnosis (represents the detection of advanced cases and is an indicator of late diagnosis). The combined use of these indicators provides a more comprehensive assessment of PHC coverage’s impact on early leprosy detection.

To understand the relationship between Primary Health Care (PHC) and Family Health Strategy (FHS) coverage and leprosy indicators, Pearson’s correlation and Prais–Winsten Linear Regression were applied. The correlation analysis assessed the degree of association between APS coverage and leprosy indicators, while trend analysis allowed for the identification of variations over time.

Pearson’s correlation is a statistical method used to measure the strength and direction of the linear association between two variables. Pearson’s correlation coefficient, represented by “r”, ranges from −1 to +1, where a positive correlation (r > 0) indicates that as one variable increases, the other tends to increase as well. For example, a positive correlation between FHS coverage and the leprosy detection rate would suggest that increased FHS coverage is associated with increased case detection. A negative correlation (r < 0) indicates that as one variable increases, the other tends to decrease. A negative correlation between PHC coverage and the detection rate of new leprosy cases with grade 2 disability, for example, would suggest that greater coverage is associated with a lower proportion of advanced cases, possibly due to early detection. A non-existent correlation (r ≈ 0) indicates that there is no apparent linear relationship between the variables analyzed, suggesting that variation in one is not associated with variation in the other. In this study, Pearson’s correlation was used to investigate the relationship between PHC and FHS coverage variables and leprosy indicators.

Prais–Winsten Linear Regression is a method for analyzing trends in time series, especially when you want to correct the autocorrelation of residuals, a common problem in time series data. This technique was applied to identify temporal trends in leprosy indicators and in PHC and FHS coverage from 2010 to 2020.

In trend analysis, three main patterns can be observed. An increasing trend indicates that the indicator or coverage variable has increased over time. For example, an upward trend in FHS coverage suggests an expansion of services over the years. A downward trend indicates that the indicator or coverage variable has decreased over time. A downward trend in the detection rate of grade 2 leprosy (G2D), for example, could indicate an improvement in early diagnosis. A stationary trend indicates that the indicator or coverage variable has remained relatively stable over time, with no significant changes.

This method made it possible to identify and evaluate changes in the trends of each variable over the study period, providing insight into how leprosy indicators and PHC and FHS coverage evolved in Minas Gerais.

As this study used secondary data, it was not necessary to use an informed consent form. This study followed the Brazilian rules on research ethics described in Resolution 466/2012.

## 3. Results

**Figure 1:**
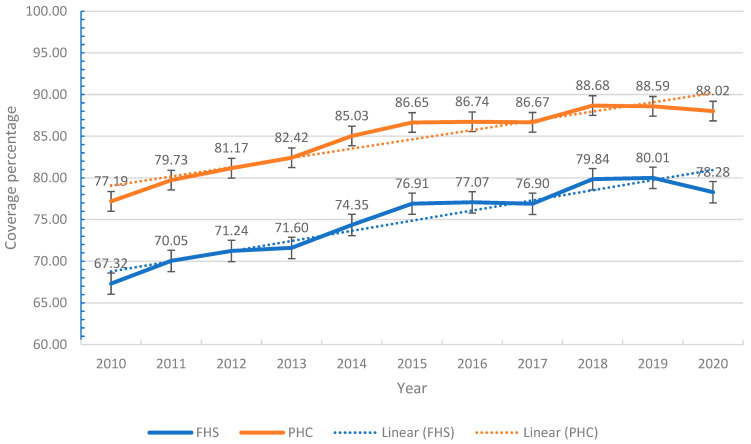
Evolution and trend of the Family Health Strategy (FHS) and Primary Health Care (PHC) coverage in Minas Gerais, 2010–2020.

The results of this study indicate that in Minas Gerais, there has been a progressive increase in the coverage of both services over time. The ESF showed the sharpest growth, rising from 67.32% in 2010 to a peak of 80.01% in 2019, followed by a slight drop to 78.28% in 2020. PHC, on the other hand, maintained high coverage throughout the period analyzed, reaching 88.02% in 2020. The linear trend indicates sustained growth, with relatively narrow confidence intervals over the years (Figure 1).

**Figure 2:**
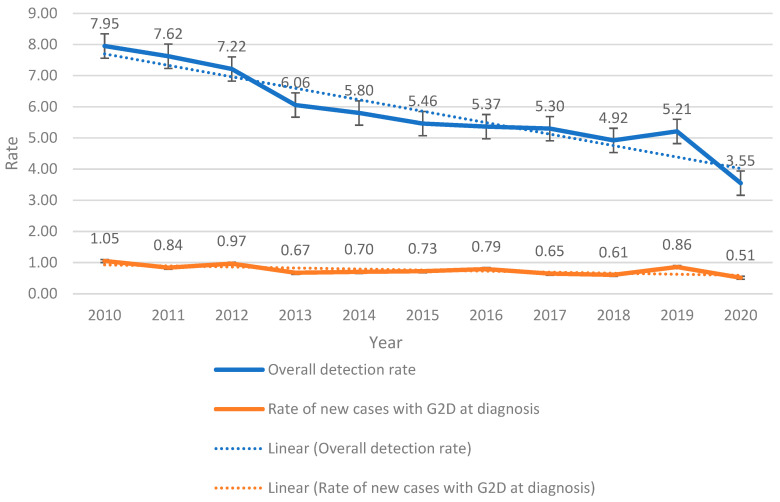
Trends in the overall leprosy detection rate and the rate of new cases diagnosed with grade 2 disability (G2D) in Minas Gerais, 2010–2020.

The overall leprosy detection rate and the rate of new cases diagnosed with grade 2 disability (G2D) between 2010 and 2020 can be seen in Figure 2. The detection rate fell significantly, from 7.95 per 100,000 inhabitants in 2010 to 3.55 per 100,000 inhabitants in 2020, accompanied by a downward trend over the period. On the other hand, the rate of new cases diagnosed with G2D remained relatively stable, with values varying between 0.51 and 1.05 per 100,000 inhabitants. Despite the overall reduction in detection, there was stability in the G2D rate (Figure 2).

These data are reiterated when considering the annual percentage change (APC). The results of this study indicate that in Minas Gerais, there was a trend towards an increase in the coverage of Primary Health Care (PHC) and the Family Health Strategy (FHS) of 1.97% and 2.21%, respectively (Table 1).

However, there was a downward trend in the rates of new leprosy cases diagnosed with grade 2 disability (G2D) and in the overall leprosy detection rate, with APCs of −6.38% and −7.62%. This behavior also suggests a significant reduction in case detection.

The correlation analysis at the state level showed that both PHC and FHS coverage had a negative association with leprosy detection rates and cases with grade 2 disability. The correlation coefficients were significant, with values of −0.7 and −0.9 between PHC and G2D and between PHC and general detection, and −0.6 and −0.9 between FHS and these same indicators, respectively, suggesting a robust inverse relationship between the expansion of health coverage and leprosy indicators.

In a regional analysis of the micro-regions of Minas Gerais, the trend in PHC and FHS coverage varied, with 71 and 72 micro-regions, respectively, showing increasing trends, while in 19 (PHC) and 18 (FHS), the trend was stationary, and none showed a decreasing trend (Table 2).

With regard to the rates of new leprosy cases with G2D and the overall detection rate, 42 and 65 micro-regions showed a downward trend, while 48 and 23 remained stationary.

The correlations between leprosy coverage and indicators in the micro-regions also showed variations. Positive correlations were found between PHC and G2D in 18 micro-regions and between PHC and general detection in 15; the FHS showed a positive correlation with G2D and general detection in 19 and 15 micro-regions, respectively. On the other hand, 41 and 55 micro-regions showed negative correlations between PHC and the same indicators, and 43 and 56 micro-regions showed negative correlations for the FHS. The absence of a correlation was observed in a significant number of micro-regions, demonstrating that the relationship between coverage and detection varies between different regional contexts.

When detailing the levels of correlation between Primary Health Care (PHC) and Family Health Strategy (FHS) coverage and leprosy indicators in the micro-regions of Minas Gerais, we see a diversity of intensities, reflecting the complexity of the relationship between health coverage and leprosy detection (Table 3).

For the relationship between PHC coverage and the rate of new leprosy cases with grade 2 disability (G2D), weak and very weak positive and negative correlations prevailed. A moderate positive correlation was identified in only one micro-region, while a weak positive correlation appeared in four micro-regions, and a very weak positive correlation appeared in thirteen. There were no records of a strong or perfect positive correlation. On the other hand, negative correlations were found in 41 micro-regions, 15 with a very weak negative correlation, 12 with a weak negative correlation, and 10 with a moderate negative correlation. Only four micro-regions showed a strong negative correlation, and none showed a perfect correlation.

When analyzing the correlation between PHC coverage and the overall leprosy detection rate, a similar pattern was observed. Most micro-regions showed weak and very weak correlations, with only one micro-region having a moderate positive correlation, five having a weak positive correlation, and nine having a very weak positive correlation. There was no significant correlation in 18 micro-regions. With regard to negative correlations, 14 micro-regions had a very weak correlation, 12 had a weak negative correlation, 13 had a moderate negative correlation, and 14 had a strong negative correlation. Two locations showed a perfect negative correlation.

When looking at the relationship between FHS coverage and the rate of cases with G2D, there was a slight variation compared to PHC coverage. In this case, one micro-region had a moderate positive correlation, four had a weak positive correlation, and fourteen had a very weak positive correlation. No strong or perfect positive correlations were observed. On the other hand, negative correlations were more prevalent, with 16 micro-regions showing a very weak negative correlation, 16 showing a weak correlation, 9 showing a moderate correlation, and 2 showing a strong negative correlation, with no perfect negative correlations recorded.

Finally, the correlation between FHS coverage and the overall leprosy detection rate reflected a composition of varying intensities. Three micro-regions showed a moderate positive correlation, two showed a weak positive correlation, and ten showed a very weak positive correlation. No significant correlation was observed in 17 micro-regions. As for negative correlations, 14 micro-regions had a very weak negative correlation, 12 showed a weak correlation, 16 showed a moderate correlation, and 13 showed a strong negative correlation. Only one micro-region showed a perfect negative correlation between FHS coverage and the overall detection rate.

This heterogeneous distribution of correlations in intensity and direction between PHC, the FHS, and leprosy indicators in Minas Gerais shows the complexity of the relationship between Primary Health Care coverage and leprosy outcomes, highlighting significant regional variations in the association between the expansion of services and the detection and severity rates of diagnosed cases.

## 4. Discussion

The results of this study indicate that the expansion of PHC and FHS coverage has not uniformly resulted in a reduction in late leprosy diagnoses. Although some regions showed a drop in the proportion of cases diagnosed with grade 2 disability, others maintained high rates of this indicator, suggesting that the expansion of coverage was not accompanied by an equivalent strengthening of early detection actions and may be influenced by structural and operational factors in the care network. Thus, the increase in coverage alone is insufficient without additional efforts in early detection and monitoring.

This finding contradicts the initial hypothesis that increased coverage would be associated with improvements in the early identification of leprosy. The literature confirms this complexity, suggesting that the impact of primary health services on neglected diseases, such as leprosy, depends on a series of structural and contextual factors, which vary according to the region and the level of social and economic development [7,8].

Previous studies suggest that the FHS plays a fundamental role in promoting the early diagnosis of infectious diseases through local actions, continuous surveillance, and strengthening ties with communities [8,9,10]. However, there is evidence that this structure faces difficulties in fully meeting demand in leprosy-endemic regions, where stigmatization and low access to resources limit the scope of health actions [7,11]. The mere expansion of the FHS, although necessary, is insufficient to meet the challenges posed by the diagnosis and monitoring of leprosy, and technical improvement and the adaptation of policies to regional and cultural specificities are essential [4]. In the context of Minas Gerais, the lack of a significant correlation between the expansion of coverage and the improvement in detection indicators suggests that the services, although expanded, do not have the necessary conditions to effectively identify and monitor cases.

The persistence of late diagnoses, evidenced by the considerable proportion of cases with grade 2 disability, reinforces the idea that structural barriers, such as inadequate training of health professionals and the scarcity of resources in the units, compromise the potential of the FHS and PHC to respond effectively to the problem [12]. In other endemic regions, researchers have argued that in order for primary health services to be truly effective in combating leprosy, it is essential to implement periodic training to improve the early diagnosis and continuous monitoring of patients [13]. In addition, the effectiveness of the services depends on strategies that integrate active detection with regular follow-up and a reduction in the stigma associated with leprosy, especially in rural and hard-to-reach areas [7,11].

The data from this study also revealed a notable heterogeneity in the correlations between health coverage and leprosy indicators in different micro-regions of Minas Gerais, which reinforces the need for a more detailed and contextualized look at the problem. Some micro-regions showed a weak or moderate positive correlation, but most showed no significant correlation, and some even showed a negative correlation between coverage and detection. This pattern suggests that local factors, such as transportation infrastructure, population mobility, and socioeconomic conditions, can affect the ability of health services to reach vulnerable populations and carry out early detection [14]. The urban or rural environment and the concentration of poverty are directly related to detection capacity, showing that the quantitative expansion of FHS coverage must be accompanied by qualitative strategies geared to the reality of each location [5,7].

This variability observed between the micro-regions of Minas Gerais confirms the gaps in the uniform implementation of public health policies and emphasizes that the increase in coverage alone does not guarantee the effectiveness of leprosy control services [7]. However, increased coverage represents the latent potential for combating leprosy, provided it is accompanied by appropriate strategic planning. This includes specific training for professionals, recommended at least annually, structured strategies for active case finding, and integration of services with local epidemiological surveillance. Additionally, it is essential to align these actions with the guidelines of the global, national, and state leprosy programs, ensuring that regions with low performance receive targeted interventions. Experience in other regions of Brazil shows that training community health workers and strengthening the bond between teams and the population can optimize the existing resources and reduce gaps in detection and treatment, especially in highly vulnerable communities [8,9]. To enhance the effectiveness of these strategies, structured training cycles for health professionals are recommended, at least twice a year, focusing on clinical diagnosis, investigation of contacts, and early recognition of neural impairment. In addition, strengthening active case finding through household visits and integrating these efforts with community health education can increase detection rates and improve treatment adherence.

In the context of this study’s central question—“Does the coverage of Primary Health Care and the Family Health Strategy influence the detection of new leprosy cases in different regions of Minas Gerais?”—the results indicate that the influence of these structures is limited and strongly conditioned by structural, regional, and cultural factors.

The relationship between the expansion of PHC and the detection of leprosy is not linear and can be influenced by factors such as geographical access, professional training, and socio-cultural barriers. Previous studies indicate that strengthening PHC can contribute to the early detection of infectious diseases, but leprosy has particularities that make this direct relationship difficult since the late detection of leprosy is often associated with difficulties in accessing health services and gaps in the training of primary care professionals [15]. Thus, the long incubation period of the disease, the stigma associated with diagnosis, and the need for specific training to assess subtle clinical signs are factors that may explain why the expansion of PHC and FHS coverage has not resulted in a uniform improvement in leprosy indicators [9]. This finding reinforces the need to invest not only in PHC coverage but also in training the health team and structuring active surveillance strategies.

In this sense, the challenges in the early detection of leprosy are directly related to the organization of health services and the qualification of PHC teams. The persistence of cases diagnosed with grade 2 physical disability at the time of diagnosis reinforces that structural barriers and difficulties in the line of care still have an impact on the early detection of the disease.

Similar studies identified three categories of factors associated with delayed diagnosis: structural, organizational, and intermediate factors [15]. Structural factors include logistical barriers, financial difficulties, and geographical limitations that restrict access to services. In addition, problems in the organization of referral services and a shortage of trained professionals also compromise early detection. At the intermediate level, this study showed that leprosy continues to be underdiagnosed, resulting in multiple consultations before a case is confirmed [15].

The convergence of these findings reinforces the need to strengthen PHC, not only in terms of coverage but also in terms of qualifying teams for the early recognition of leprosy. Strategies that ensure the sustainability of disease control actions within integrated health services are fundamental to reducing delays in diagnosis and minimizing associated disabilities.

Therefore, by investigating the relationship between PHC and FHS coverage and leprosy indicators, this study confirms that although expanding coverage is important, it must be part of a broader approach that considers the qualification of professionals and the specificities of the communities served, as well as active detection strategies and stigma control [4,11]. Expanding coverage without addressing gaps in service effectiveness—such as the limited adoption of active case finding and inconsistent application of WHO guidelines—may result in an inefficient allocation of resources. Therefore, it is crucial to evaluate not only the reach of the FHS but also its capacity to provide effective leprosy control interventions.

## 5. Conclusions

The findings of this study indicate that the expansion of PHC and FHS coverage in Minas Gerais was not directly associated with a homogeneous reduction in the late diagnosis of leprosy. There was a reduction in the rate of new cases with grade 2 disability in some regions, but others maintained high rates of this indicator. Furthermore, there was no strong correlation between PHC coverage and improvements in leprosy indicators. Thus, the persistence of high rates of cases with grade 2 disability in some regions suggests that increased coverage alone does not guarantee the expected impact, i.e., it is not enough to guarantee effective early detection. It is therefore necessary to strengthen professional training strategies, intensify the active search for cases, and integrate new approaches in order to optimize the response of the leprosy program.

Studies suggest that an integrated approach, which includes health education, community participation, and support for professionals, can increase the effectiveness of the FHS in reducing the burden of neglected diseases [13]. In summary, expanding coverage is a necessary but not sufficient condition for improving leprosy indicators, and it is essential that interventions consider local realities and promote health care that is inclusive, empowered, and culturally adapted to the populations served.

## Figures and Tables

**Table 1:** Trends and correlations of health and epidemiological indicators of leprosy in Minas Gerais, 2010 to 2020.

Trend *	**PHC Coverage**	**FHS Coverage**	**Rate of New Cases with G2D at Diagnosis**	**Overall Detection Rate**
Minas Gerais	Growing	Growing	Decreasing	Decreasing
	APC 1.97	APC 2.21	APC −6.38	APC −7.62
Correlation **	**PHC X G2D**	**PHC X Overall Detection Rate**	**FHS X G2D**	**FHS X Overall Detection Rate**
Minas Gerais	Negative correlation	Negative correlation	Negative correlation	Negative correlation
	−0.7	−0.9	−0.6	−0.9

Prepared for the purposes of this study. Legend: PHC—Primary Health Care; FHS—Family Health Strategy; G2D—rate of new cases with grade 2 disability at diagnosis. Note: * Praiss–Winsten Linear Regression; ** Pearson correlation.

**Table 2:** Trends and correlations of health and epidemiological indicators of leprosy in Minas Gerais by health micro-region (n), 2010 to 2020.

Trend *	**PHC Coverage**	**FHS Coverage**	**Rate of New Cases with G2D at Diagnosis**	**Overall Detection Rate**
Growing	71	72	0	2
Decreasing	0	0	42	65
Stationary	19	18	48	23
Correlation **	**PHC X G2D**	**PHC X Overall Detection Rate**	**FHS X G2D**	**FHS X Overall Detection Rate**
Positive	18	15	19	15
None	24	18	21	17
Negative	41	55	43	56

Prepared for the purposes of this study. Legend: PHC—Primary Health Care; FHS—Family Health Strategy; G2D—rate of new cases with grade 2 disability at diagnosis. Note: * Praiss–Winsten Linear Regression; ** Pearson correlation.

**Table 3:** Breakdown of correlations of health and epidemiological indicators of leprosy in Minas Gerais by health micro-region (n), 2010 to 2020.

Correlation *	PHC X G2D	PHC X Overall Detection Rate	FHS X G2D	FHS X Overall Detection Rate
Perfect positive correlation	0	0	0	0
Strong positive correlation	0	0	0	0
Moderate positive correlation	1	1	1	3
Weak positive correlation	4	5	4	2
Very weak positive correlation	13	9	14	10
No significant correlation	24	18	21	17
Very weak negative correlation	15	14	16	14
Weak negative correlation	12	12	16	12
Moderate negative correlation	10	13	9	16
Strong negative correlation	4	14	2	13
Perfect negative correlation	0	2	0	1

Prepared for the purposes of this study. Legend: PHC—Primary Health Care; FHS—Family Health Strategy; G2D—rate of new cases with grade 2 disability at diagnosis. Note: * Pearson correlation.

## Data Availability

The data presented in this study were obtained from public domain resources, including the Notifiable Diseases Information System (SINAN), extracted using the Tabwin software, and the EGESTOR-AB portal of the Department of Informatics of the Unified Health System (DATASUS). Both datasets are publicly accessible through the DATASUS portal at [https://datasus.saude.gov.br/ 6 March 2025].

## Abbreviations

The following abbreviations are used in this manuscript:

PHC	Primary Health Care
FHS	Family Health Strategy
SINAN	Notifiable Diseases Information System
DATASUS	Department of Informatics of the Unified Health System
G2D	grade 2 disability
APC	annual percentage change
